# Combinational Reasoning of Quantitative Fuzzy Topological Relations for Simple Fuzzy Regions

**DOI:** 10.1371/journal.pone.0117379

**Published:** 2015-03-16

**Authors:** Bo Liu, Dajun Li, Yuanping Xia, Jian Ruan, Lili Xu, Huanyi Wu

**Affiliations:** 1 Faculty of Geomatics, East China Institute of Technology, 330013, Nanchang, PR, China; 2 State Key Laboratory of Information Engineering in Surveying, Mapping and Remote Sensing, Wuhan University, 430079, Wuhan, PR, China; Southwest University, CHINA

## Abstract

In recent years, formalization and reasoning of topological relations have become a hot topic as a means to generate knowledge about the relations between spatial objects at the conceptual and geometrical levels. These mechanisms have been widely used in spatial data query, spatial data mining, evaluation of equivalence and similarity in a spatial scene, as well as for consistency assessment of the topological relations of multi-resolution spatial databases. The concept of computational fuzzy topological space is applied to simple fuzzy regions to efficiently and more accurately solve fuzzy topological relations. Thus, extending the existing research and improving upon the previous work, this paper presents a new method to describe fuzzy topological relations between simple spatial regions in Geographic Information Sciences (GIS) and Artificial Intelligence (AI). Firstly, we propose a new definition for simple fuzzy line segments and simple fuzzy regions based on the computational fuzzy topology. And then, based on the new definitions, we also propose a new combinational reasoning method to compute the topological relations between simple fuzzy regions, moreover, this study has discovered that there are (1) 23 different topological relations between a simple crisp region and a simple fuzzy region; (2) 152 different topological relations between two simple fuzzy regions. In the end, we have discussed some examples to demonstrate the validity of the new method, through comparisons with existing fuzzy models, we showed that the proposed method can compute more than the existing models, as it is more expressive than the existing fuzzy models.

## Introduction

Geographical information sciences (GIS) commonly deal with geographical phenomena modeled by crisp points, lines, and regions, which are clearly defined or have crisp boundaries. However, geospatial data are always uncertain or fuzzy due to inaccurate data acquisition, incomplete representation, dynamic change, and the inherent fuzziness of geographical phenomena itself. In GIS, many studies have been devoted to modeling topological relations, specifically the modeling of quantitative fuzzy topological relations between simple spatial objects. Topology is a fundamental challenge when modeling spatial relations in geospatial data that includes a mix of crisp, fuzzy and complex objects. Two mechanisms, the formalization and reasoning of topological relations, have become popular in recent years to gain knowledge about the relations between these objects at the conceptual and geometrical levels. These mechanisms have been widely used in spatial data query [1–2], spatial data mining [3], evaluation of equivalence and similarity in a spatial scene [4], and for consistency assessment of the topological relations of multi-resolution spatial databases [5–7]. Dilo et al. [8] defined several types and operators for modeling spatial data systems to handle fuzzy information. Shi et al. [9] and Liu et al. [10] developed a new object extraction and classification method based on fuzzy topology.

However, the uncertain topological relations themselves must be modeled due to the existence of indeterminate and uncertain boundaries between spatial objects in GIS. Fuzzy topology theory can potentially be applied to the modeling of fuzzy topological relations among spatial objects.

To date, many models have been designed to formalize topological relations between simple fuzzy objects, they can be implemented on spatial databases at less cost than other uncertainty models and are useful when managing, storing, querying, and analyzing uncertain data. For example, Egenhofer and Franzosa [5, 11] and Winter [12] modeled the topological relations between two spatial regions in two dimensional space (2-D) based on the 4-intersection model and ordinary point set theory. Li et al. [13], Long and Li [14] produced a Voronoi-based 9-intersection model based on Voronoi diagrams. Cohn et al. [15, 16] discovered forty-six topological relations between two regions with indeterminate boundaries based on the region connection calculus (RCC) theory [17]. Clemmentini and Di Felice [18–20] used extended 9-intersection model to classify forty-four topological relations between simple regions with broad boundaries. The extended 9-intersection model substantially agrees with the RCC model, though the former removes two relations considered as invalid in the geographical environment. The extended 9-intersection model can be extended to represent topological relations between objects with different dimensions, like regions and lines, while the RCC model can only be applied to relations between regions. Tang and Kainz [21], Tang et al. [22], Tang [23] applied fuzzy theory and a 9-intersection matrix, discovered forty-four topological relations between two simple fuzzy regions. Shi and Liu [24] discussed fuzzy topological relations between fuzzy spatial objects based on the theory of fuzzy topology. Du et al. [25, 26] proposed computational methods for fuzzy topological relations description, as well as a new fuzzy 9-intersection model. These models provided a framework to conceptually describe the topological relations between two regions and can be considered as an extension of the crisp case. In order to define and describe spatial relations qualitatively and to compute the fuzzy topological relations quantitatively, Liu and Shi [27, 28], Shi and Liu [29] defined a computational fuzzy topology to compute the interior, boundary, and exterior parts of spatial objects, and based on these definitions, Liu and Shi [28] proposed a computational 9-intersection model, and discovered sixteen topological relations between simple fuzzy region and line segment; forty-six topological relations between simple fuzzy line segments; three topological relations between simple fuzzy region and fuzzy point; and three topological relations between simple fuzzy line segment and fuzzy point, but it did not give the topological relations between two simple fuzzy regions, and it did not compute the topological relations between one simple fuzzy spatial region and one simple crisp spatial region.

To obtain the quantitative fuzzy topological relations between simple fuzzy regions in GIS, based on the previous researches, this paper develops a new method of describing quantitative fuzzy topological relations for simple fuzzy regions. The new method not only computes the topological relations between one simple fuzzy spatial region and one simple crisp spatial region, but also computes the topological relations between two simple fuzzy regions.

The remainder of this paper is organized as follows. In section 2, some basic concepts of fuzzy topology, computational fuzzy topology and the definitions of simple fuzzy spatial objects in GIS are reviewed; In Section 3, the new definitions of simple fuzzy spatial objects are introduced, and we proposed a new combinational reasoning method of quantitative fuzzy topological relations for simple fuzzy regions; In Section 4, by comparing the proposed method with existing fuzzy models, we demonstrated that it can make finer distinctions and is more expressive; Finally, some conclusions are drawn in Section 5.

## A Brief Summary of Computational Fuzzy Topology

In this section, coherent fuzzy topologies, induced by interior and closure operators [27–30], are reviewed. Mathematically, point set topology is the fundamental theory for modeling topological relations between crisp spatial objects in a GIS. By extension, fuzzy topology is a generalization of ordinary topology that introduces the concept of membership value and can be adopted for modeling topological relations between spatial objects with uncertainties. Zadeh [31] introduced the concept of fuzzy sets, and fuzzy set theory. Fuzzy topology was further developed based on the fuzzy sets [30, 32–34]. Liu and Shi [27, 28] and Shi and Liu [29] define a computational fuzzy topology to compute the interior, boundary and exterior of spatial objects. The computation is based on two operators, the interior operator and the closure operator. Each interior operator corresponds to one fuzzy topology and that each closure operator also corresponds to one fuzzy topology [30]. The research detailed in this paper extends this work by defining fuzzy spatial objects. However, it is important to review basic concepts in fuzzy set theory as well as simple fuzzy objects in a GIS.

### Basic concepts

We focus on the two-dimensional Euclidean plane *R* ^*2*^, with the usual distance and topology. Fuzzy topology is an extension of ordinary topology that fuses two structures, order structure and topological structure. Furthermore, the fuzzy interior, boundary, and exterior play an important role in the uncertain relations between GIS objects. Therefore, Shi and Liu [29] gave those items a very clean concept and the definition on fuzzy topological space.

In this section, we present the basic definitions for fuzzy sets and fuzzy mapping briefly, details on these definitions are given in related references [27–30, 32]. Fuzzy sets, open fuzzy sets, and closed fuzzy sets are the basic elements of fuzzy topology. The following are several definitions of fuzzy sets.

**Definition 1 (Fuzzy subset)**. Let *X* be a non-empty ordinary set and *I* be the closed interval [0, 1].

An *I-*fuzzy subset on *X* is a mapping (called the membership function of *A*) *μ*_*A*_ : *X* → *I*, i.e., the family of all the [0,1]-fuzzy or *I-*fuzzy subsets on *X* is just *I* ^*X*^ consisting of all the mappings from *X* to *I*. Here, *I* ^*X*^ is called an *I-*fuzzy space. *X* is called the carrier domain of each *I*-fuzzy subset on it, and *I* is called the value domain of each *I-*fuzzy subset on *X*. *A* ∈ *I* ^*X*^ is called a crisp subset on *X*, if the image of the mapping is the subset of {0,1} ⊂ *I*.

**Definition 2 (Rules of set relations)**. Let *A* and *B* be fuzzy sets in *X* with membership functions *μ*_*A*_(*x*) and *μ*_*B*_(*x*), respectively. Then,

1. *A* = *B*, *iff* *μ*_*A*_(*x*) = *μ*_*B*_(*x*) *for all x in X*;
2. *A* ≤ *B*, *iff* *μ*_*A*_(*x*) ≤ *μ*_*B*_(*x*) *for all x in X*;
3. *C* = *A* ∨ *B*, *iff μ*_*C*_(*x*) = max[*μ*_*A*_(*x*), *μ*_*B*_(*x*)] *for all x in X*;
4. *D* = *A* ∧ *B*, *iff μ*_*D*_(*x*) = min[*μ*_*A*_(*x*), *μ*_*B*_(*x*)] *for all x in X*;
5. *E = A* ∖ *B*, *iff μ*_*E*_(*x*) = 1− *μ*_*A*_(*x*) *for all x in X*.

The fuzzy interior, boundary, and exterior play an important role in the uncertain relations between GIS spatial objects [29]. In the following, we will introduce the concept of fuzzy topology, which will be a basis for the description of uncertain relations between simple spatial objects in GIS. Furthermore, and the following are the commonly used definitions of fuzzy topological space, fuzzy interior, fuzzy closure, fuzzy complement, and fuzzy boundary [30].

**Definition 3 (Fuzzy topological space)**. Let *X* be a non-empty ordinary set and *I* = [0, 1], *δ* ⊂ *I* ^*X*^. *δ* is called an *I*-fuzzy topology on *X*, and (*I* ^*X*^, *δ*) is called an *I*-fuzzy topological space (*I*-fts), if *δ* satisfies the following conditions:

1. 0,1 ∈*δ*;
2. *If A*, *B* ∈ *δ*, *then A* ∧ *B* ∈ *δ*;
3. *Let*{*A*_*i*_ : *i* ∈ *J*}⊂*δ*, *whereJis an index set*, *then* ∨_*i*∈*J*_ *A*_*i*_ ∈ *δ*.

Where, 0 ∈ *δ* means the empty set and 1 ∈ *δ* means the whole set *X*. The elements in *δ* are called open elements and the elements in the complement of *δ* are called closed elements, and the set of the complement of open set is denoted by $\delta^{\prime }$.

**Definition 4 (Interior and closure)**. For any fuzzy set *A* ∈ *I* ^*X*^, the interior of *A* is defined as the join of all the open subsets contained in *A*, denoted by *A* ^*∘*^, and the closure of *A* as the meeting of all the closed subsets containing *A*, denoted by $\bar{A}$.

**Definition 5 (Fuzzy complement)**. For any fuzzy set *A*, we defined the complements of *A* by *A* ^*C*^(*x*) = 1 − *A*(*x*), denoted by *A* ^*C*^.

**Definition 6 (Fuzzy boundary)**. The boundary of a fuzzy set *A* is defined as $\partial A=\bar{A}\wedge \bar{A^{C}}$.

**Definition 7 (Closure operator)**. An operator *α* : *I* ^*X*^ → *I* ^*X*^ is a fuzzy closure operator if the following conditions are satisfied:

1. *α*(0) = 0;
2. *α*(*A*) ≤ *A*, *for all A* ∈ *I* ^*X*^;
3. *α*(*A* ∨ *B*) = *α*(*A*) ∨ *α*(*B*);
4. *α*(*α*(*A*)) = *α*(*A*), *for all A* ∈ *I* ^*X*^.

**Definition 8 (Interior operator)**. An operator *α* : *I* ^*X*^ → *I* ^*X*^ is a fuzzy interior operator if the following conditions are satisfied:

1. *α*(1) = 1;
2. *A* ≤ *α*(*A*), *for all A* ∈ *I* ^*X*^;
3. *α*(*A* ∧ *B*) = *α*(*A*) ∧ *α*(*B*);
4. *α*(*α*(*A*)) = *α*(*A*), *for all A* ∈ *I* ^*X*^.

**Definition 9 (Interior and closure operators)**. For any fixed *α* ∈ [0,1], both operators, interior and closure, are defined as $A_{\alpha }(x)=\left\{ \begin{array}{l} A(x)\begin{array}{ccc} & if & A(x)>\alpha \end{array}\\ 0\begin{array}{cccc} & & if & A(x)\leq \alpha \end{array} \end{array}\right.$ and $A^{1-\alpha }(x)=\left\{ \begin{array}{l} 1\begin{array}{ccc} & if & A(x)\geq 1-\alpha \end{array}\\ A(x)\begin{array}{cccc} & & if & A(x)<1-\alpha \end{array} \end{array}\right.$, respectively. And induced an *I-*fuzzy topology (*X*, *τ*_*α*_, *τ* ^1−*α*^) in *X*, where *τ*_*α*_ = {*A*_*α*_ : A ∈ *I* ^*X*^} is the open sets and *τ* ^1−*α*^ = {*A* ^1−*α*^ : A ∈ *I* ^*X*^} is the closed sets. The elements in *τ*_*α*_ and *τ* ^1−*α*^ satisfy the relations (*A*_*α*_)^*C*^ = (*A* ^*C*^) ^1−*α*^, for all fuzzy set *A*, i.e., the complement of the element in the *τ*_*α*_ closed set. Details on how these two operators can induce a coherent *I*-fuzzy topology are given in Liu and Shi [27].

To study topological relations, it is essential to firstly understand the properties of a fuzzy mapping, especially the homeomorphic mapping since topological relations are invariant in homeomorphic mappings. The following section presents a number of definitions related to fuzzy mapping.

Let *I* ^*X*^, *I* ^*Y*^ be *I*-fuzzy spaces, *f* : *X* → *Y* an ordinary mapping. Based on *f* : *X* → *Y*, define *I*-fuzzy mapping *f* ^→^ : *I* ^*X*^ → *I* ^*Y*^ and its *I*-fuzzy reverse mapping *f* ^→^ : *I* ^*X*^ → *I* ^*Y*^ by

$$f^{\to }\;:\;I^{X}\;\to \;I^{Y},\;f^{\to }(A)(y)=\{\begin{array}{l} V\left\{A(x)\right\},\begin{array}{ccc} if & x\in f^{-1}(y) & \end{array}\\ 0\begin{array}{ccc} \begin{array}{ccc} & & otherwise \end{array} & & \end{array} \end{array},\;\forall A\;\in \;I^{X},\;\forall y\;\in \;Y\;$$

*f* ^←^ : *I* ^*Y*^ → *I* ^*X*^, *f* ^←^ (*B*)(*x*) = *B*(*f*(*x*)), ∀*B* ∈ *I* ^*Y*^, ∀*x* ∈ *X* While *I* = [0,1].

Let (*I* ^*X*^, *δ*), (*I* ^*Y*^, *δ*) be *I*-fts’s, *f* ^→^ : (*I* ^*X*^, *δ*) →(*I* ^*Y*^, *μ*) is called *I*-fuzzy homeomorphism, if it is bijective continuous and open [30]. One important theorem to check an *I*-fuzzy homeomorphism is that, as proved by Shi and Liu [29]. Let *A* ∈ *I* ^*X*^, *B* ∈ *I* ^*Y*^, let (*I* ^*X*^, *δ*), (*I* ^*Y*^, *δ*) be *I*-fts’s induces by interior operator and closure operators. Then *f* ^→^ : (*I* ^*X*^, *δ*) → (*I* ^*Y*^, *μ*) is an *I*-fuzzy homeomorphism if and only if *f* : *X* → *Y* is bijective mapping. Meanwhile, for the topology induced by these two operators, for checking homeomorphic map, actually, we only have to check whether it is one-one corresponding between domain and range only.

### Modeling simple fuzzy spatial objects in GIS

Geometrically, GIS features can be classified as points, lines, and regions, and within the framework of crisp objects modeling in GIS, we first need to model simple objects before we model the topological relations between the objects. Similarly, within the framework of fuzzy objects modeling in GIS, we first need to model simple fuzzy objects before we can model the fuzzy topological relations between the objects [19]. Many researchers also have been working on modeling fuzzy topological relations and simple fuzzy objects [21, 27–29]. For example, Tang and Kainz [21] defined a simple fuzzy region based on fuzzy topology; Liu and Shi [27–29] have provided definitions of simple fuzzy points, simple fuzzy line segments and simple fuzzy regions.

In this research, the mentioned fuzzy definitions of the basic elements(simple fuzzy points, lines, and regions) in GIS are summarized briefly as follows, details on these definitions are given in related references [27–29].

**Fig 1:**
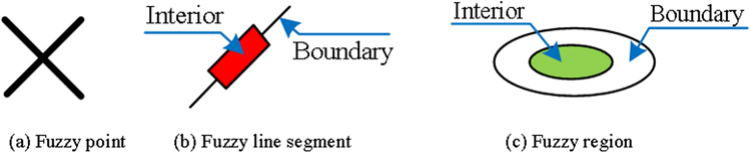
(a) Fuzzy point for given *α*; (b) Fuzzy line segment for given *β*; (c) Fuzzy region for given *γ* [28].

**Definition 10 (fuzzy point**, Fig. 1(a)). An *I*-fuzzy point on *X* is an *I*-fuzzy subset *X*_*α*_ ∈ *I* ^*X*^, defined as: $x_{\alpha }(y)=\left\{ \begin{array}{l} \begin{array}{ccc} \alpha & if & y=x \end{array}\\ \begin{array}{ccc} 0 & otherwise & \end{array} \end{array}\right.$.

**Definition 11 (Simple fuzzy line)**. A fuzzy subset in *X* is called a simple fuzzy line (*L*), if *L* is a supported connected line in the background topology (i.e., a crisp line in the background topology).

**Definition 12 (Simple fuzzy line segment**, Fig. 1(b)). The simple fuzzy line segment (*L*) is a fuzzy subset in *X* with: 1) for any *β* ∈ (0,1), the fuzzy line *L*_*β*_ is a supported connected line segment (i.e., a crisp line segment in the background topology) in the background topology and 2) ∂*L* (= (*L*_*β*_)^*C*^ ∧ *L* ^1−*β*^)) has at most two supported connected components.

**Definition 13 (Simple fuzzy region**, Fig. 1(c)). A simple fuzzy region is a fuzzy region in a polygon feature *X* which: 1) for any *γ* ∈ (0,1), the fuzzy set *A*_*γ*_ and ∂*A*(*A*_*γ*_)^*C*^ ∧ *L* ^1−*γ*^ are two supported connected regular bounded open sets in the background topological space; 2) in the background topological space, any outward normal from *Supp*(*A*_*γ*_) must meet *Supp*(∂*A*) and have only one component.

On the basic definitions of simple fuzzy points, line segments and regions, Shi and Liu [29] provide an example of computing the interior, boundary, and exterior of spatial objects for different *α* values, and the interior, boundary, and exterior of spatial objects were confirmed for each given *α* value. Based on the fuzzy objects’ definitions, Liu and Shi [28] proposed a new 3 × 3 integration model to compute sixteen topological relations between a simple fuzzy region to a simple fuzzy line segment, three topological relations between a simple fuzzy region to a simple fuzzy point, forty-six topological relations between a simple fuzzy line segment and a simple fuzzy line segment, and three topological relations between a simple fuzzy line segment and a simple fuzzy point. However, the relations between two simple fuzzy regions were not computed, and the element *f*_*X*_ *A* ∧ *BdV* of the 3 × 3 integration model is the ratio of the area (or volume) of meet of two fuzzy spatial objects in a join of two simple spatial object (here a join of two fuzzy objects means ‘union’ of two fuzzy objects; meet of two fuzzy objects means ‘intersection’ of two fuzzy objects [28]), and was difficult to describe the topological relations between simple crisp spatial regions and simple fuzzy spatial regions.

Based on existing related studies, in next section, we will discuss the newly combinational reasoning method to compute the topological relations between simple fuzzy regions.

## Combinational Reasoning of Quantitative Fuzzy Topological Relations for Simple Fuzzy Regions

### The basic topological relations between two simple crisp regions

**Fig 2:**
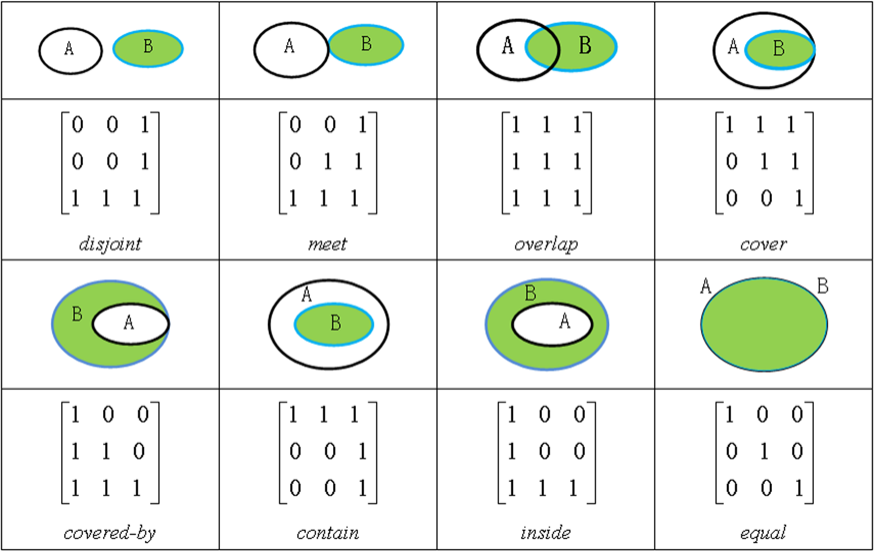
The eight basic topological relations between two simple crisp regions [36]

Many models have been designed to formalize topological relations between simple crisp objects, such as the 4-Intersection Model (4IM) [11, 35], the 9-Intersection Model (9IM) [36], Region Connection Calculus (RCC) theory [17], and the Voronoi-based 9-Intersection Model (V9I) [13, 14]. All of the existing models can determine the eight basic topological relations possible for 2-dimensional simple crisp regions in the physical world: *disjoint*, *meet*, *overlap*, *cover*, *contain*, *equal*, *inside*, *and covered-by*, respectively as seen in Fig. 2. In point set topology, the 4IM, 9IM, and V9I models divide a simple crisp region into three parts: interior (*A* ^*∘*^), boundary (*∂A*), and exterior $\left(A^{-}/A^{V}\right)$. In this paper, we used 9IM (as Eq. (1)) to compute the eight basic topological relations of simple crisp regions.

$$R_{9}\left(A,B\right)=\left[\begin{array}{l} \begin{array}{cc} \begin{array}{cc} A^{{}^{\circ}}\cap B^{{}^{\circ}} & A^{{}^{\circ}}\cap \partial B \end{array} & A^{{}^{\circ}}\cap B^{-} \end{array}\\ \begin{array}{ccc} \begin{array}{l} \partial A\cap B^{{}^{\circ}}\\ A^{-}\cap B^{{}^{\circ}} \end{array} & \begin{array}{l} \partial A\cap \partial B\\ A^{-}\cap \partial B \end{array} & \begin{array}{l} \partial A\cap B^{-}\\ A^{-}\cap B^{-} \end{array} \end{array} \end{array}\right]$$ (1)

Since the intersection of two sets can be either 0 or 1, the topological relation between two simple crisp regions as follows.

### The new definitions of simple fuzzy spatial objects

A given *α*, a simple fuzzy spatial line segment and region in GIS has a certain interior and boundary as shown in Fig. 1. We developed a new definition for a simple fuzzy spatial line segment and region by applying the definition in the previous section, and on fuzzy topological space [32], the fuzzy point definition remains the same as Definition 10.

**Fig 3:**
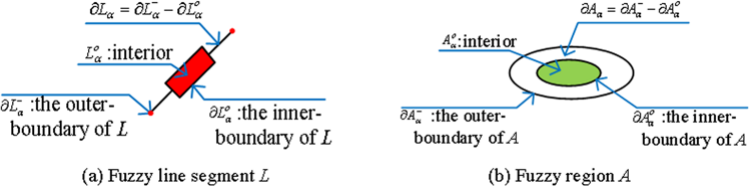
(a) The inner-outer boundary of simple fuzzy line segment *L* for given *α*; (b) the inner-outer boundary of simple fuzzy region *A* for given *α*.

**Definition 14 (inner and outer boundary of simple fuzzy line segment**, Fig. 3(a)), for given *α*, the interior and boundary of simple line segment *L(as shown in Fig. 1(b))* are confirmed, in the same way as a simple crisp line segment. Hence, we define $\partial L_{\alpha }^{-}$ as the outer-boundary of *L*, and $\partial L_{\alpha }^{o}$ as the inner-boundary of *L*, $L_{\alpha }^{o}$ as the interior of *L*, and *∂L*_*α*_ as the boundary of *L (as shown in Fig. 3(a)):* $\partial L_{\alpha }=\partial L_{\alpha }^{-}-\partial L_{\alpha }^{o}$. So, a simple fuzzy line segment *L* for given *α* can be expressed as: $L_{\alpha }=\partial L_{\alpha }^{-}\cup \partial L_{\alpha }\cup \partial L_{\alpha }^{o}\cup L_{\alpha }^{o}$.

**Definition 15 (inner and outer boundary of simple fuzzy region**, Fig. 3(b)), for a given *α*, the interior and boundary of fuzzy region *A (as shown in Fig. 1(c))* is confirmed. Just as for a crisp region, we defined $\partial A_{\alpha }^{-}$ as the outer-boundary of *A*, and $\partial A_{\alpha }^{o}$ as the inner-boundary of *A*, $A_{\alpha }^{o}$ as the interior of *A* and *∂A*_*α*_ as the boundary of *A (as shown in Fig. 3(b)):* $\partial A_{\alpha }=\partial A_{\alpha }^{-}-\partial A_{\alpha }^{o}$. So the fuzzy region *A* for given *α* can be expressed as: $A_{\alpha }=\partial A_{\alpha }^{-}\cup \partial A_{\alpha }\cup \partial A_{\alpha }^{o}\cup A_{\alpha }^{o}$. Meanwhile, $\partial A_{\alpha }^{-}$ and $\partial A_{\alpha }^{o}$ can be considered as two simple crisp regions.

Based on the above definitions, 1) to a simple fuzzy line segment *L* for given *α*, if $\partial L_{\alpha }^{-}=\partial L_{\alpha }^{o}$, that is *∂L*_*α*_ = 0, and $L=L_{\alpha }=\partial L_{\alpha }^{-}\cup L_{\alpha }^{o}$, it means *L* is a simple crisp line segment; 2) to a fuzzy region *A* for given *α*, if $\partial A_{\alpha }^{-}=\partial A_{\alpha }^{o}$, that is *∂A*_*α*_ = 0, $A=A_{\alpha }=\partial A_{\alpha }^{-}\cup A_{\alpha }^{o}$, it means *A* is a simple crisp region.

On the basis of these definitions, we will primarily focus on discussing the new combinational reasoning method of quantitative fuzzy topological relations for simple fuzzy regions in next section.

### Combinational reasoning of quantitative fuzzy topological relations for simple fuzzy regions

In this section, we will only discuss the topological relations between the simple fuzzy regions, and we will develop a new combinational reasoning method of quantitative fuzzy topological relations for simple fuzzy regions.

**Fig 4:**
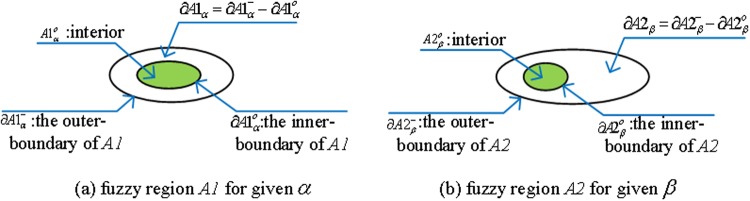
(a) Fuzzy region *A1* for given *α*; (b) Fuzzy region *A2* for given *β*.

For a fuzzy region *A1* for given *α* (Fig. 4 (a)), while the other fuzzy region *A2* for given *β* (Fig. 4 (b)), Considering the relationship between the outer-boundary ($\partial A1_{\alpha }^{-}$/$\partial A2_{\beta }^{-}$), boundary (∂*A*1_*α*_/∂*A*2_*β*_), inner-boundary ($\partial A1_{\alpha }^{o}$/$\partial A2_{\beta }^{ο}$), and interior ($A1_{\alpha }^{o}$/$A2_{\beta }^{ο}$) of the simple fuzzy region *A* and *B* (as shown in Fig. 4), their topological relations have three different situations:

- If $\partial A1_{\alpha }^{-}=\partial A1_{\alpha }^{o}$, that is ∂*A*1_*α*_ = 0, and $A1=A1_{\alpha }=\partial A1_{\alpha }^{-}\cup A1_{\alpha }^{o}$. If $\partial A2_{\beta }^{-}=\partial A2_{\beta }^{o}$, that is ∂*A*2_*β*_ = 0, and $A2=A2_{\beta }=\partial A2_{\beta }^{-}\cup A2_{\beta }^{o}$. *A1* and *A2* are simple crisp regions, the eight topological relations *R*(*A*1, *A*2) between *A1* and *A2* (as shown in Fig. 2). So, *R*(*A*1, *A*2) can be computed by 4IM [11], 9IM [36], or other existing models.
- If $\partial A1_{\alpha }^{-}=\partial A1_{\alpha }^{o}$, that is ∂A1_*α*_ = 0, and $A1=A1_{\alpha }=\partial A1_{\alpha }^{-}\cup A1_{\alpha }^{o}$. If $\partial A2_{\beta }^{-}\neq \partial A2_{\beta }^{o}$, that is *∂A*2_*β*_ ≠ 0, $A2=A2_{\beta }=\partial A2_{\beta }^{-}\cup \partial A2_{\beta }\cup \partial A2_{\beta }^{o}\cup A2_{\beta }^{o}$. *A1* is a crisp region, *A2* is a fuzzy region, and comprised of four components $\partial A2_{\beta }^{-}$, $\partial A2_{\beta }^{o}$ and $A2_{\beta }^{o}$. The topological relations between *A1* and *A2* can be described as $R(A1,A2_{\beta }^{-})$ and $R(A1,A2_{\beta }^{o})$ which can be computed by 4IM [11], 9IM [36], or other existing models.

Since all of the existing models can determine eight basic topological relations, such as{*disjoint*, *meet*, *overlap*, *cover*, *contain*, *equal*, *inside*, *and covered-by*}, each topological relation between two simple crisp regions $R(A1,A2_{\beta }^{-})$ and $R(A1,A2_{\beta }^{o})$ can be any one of the eight basic topological relations, hence in theory, $R(A1,A2_{\beta }^{-})$ and $R(A1,A2_{\beta }^{o})$ can determine 8^2^ = 64 topological relations, but only 23 basic relations for 2-dimensional simple regions in the physical world have been defined, and the relevant topological relations between *A1* and *A2* are included within the Supporting Information (S1 Table).

1. $IfR(A1,A2_{\beta }^{-})=disjoint\text{,}$ $R(A1,A2_{\beta }^{o})=disjoint\text{;}$
2. $IfR(A1,A2_{\beta }^{-})=meet\text{,}$ $R(A1,A2_{\beta }^{o})=disjoint\text{;}$
3. $IfR(A1,A2_{\beta }^{-})=overlap\text{,}$
$R(A1,A2_{\beta }^{o})=\left\{disjoint,meet,overlap,covered-by,inside\right\}\text{;}$
4. $IfR(A1,A2_{\beta }^{-})=cover\text{,}$
$R(A1,A2_{\beta }^{o})=\left\{disjoint,meet,overlap,covered-by,inside\right\}\text{;}$
5. $IfR(A1,A2_{\beta }^{-})=contain\text{,}$
$R(A1,A2_{\beta }^{o})=\left\{disjoint,meet,overlap,cover,contain,equal,inside,covered-by\right\}\text{;}$
6. $IfR(A1,A2_{\beta }^{-})=covered-by$, $R(A1,A2_{\beta }^{o})=\left\{inside\right\}$;
7. $IfR(A1,A2_{\beta }^{-})=inside\text{,}$ $R(A1,A2_{\beta }^{o})=inside\text{;}$
8. $IfR(A1,A2_{\beta }^{-})=equal\text{,}$ $R(A1,A2_{\beta }^{o})=inside\text{.}$

iii) If $\partial A1_{\alpha }^{-}\neq \partial A1_{\alpha }^{o}$, that is *∂A*1_*α*_ ≠ 0, and $A1=A1_{\alpha }=\partial A1_{\alpha }^{-}\cup \partial A1_{\alpha }\cup \partial A1_{\alpha }^{o}\cup A1_{\alpha }^{o}$. If $\partial A2_{\beta }^{-}\neq \partial A2_{\beta }^{o}$, that is *∂A*2_*β*_ ≠ 0, and $A2=A2_{\beta }=\partial A2_{\beta }^{-}\cup \partial A2_{\beta }\cup \partial A2_{\beta }^{o}\cup A2_{\beta }^{o}$. *A1* and *A2* are fuzzy regions, *A1* comprised of four components $\partial A1_{\alpha }^{-}$, *∂A*1_*α*_, $\partial A1_{\alpha }^{o}$ and $A1_{\alpha }^{o}$, meanwhile, $\partial A1_{\alpha }=\partial A1_{\alpha }^{-}-\partial A1_{\alpha }^{o}$, *A2* also comprised of four components $\partial A2_{\beta }^{-}$, *∂A*2_*β*_, $\partial A2_{\beta }^{o}$, and $A2_{\beta }^{o}$, meanwhile, $\partial A2_{\beta }=\partial A2_{\beta }^{-}-\partial A2_{\beta }^{o}$. The topological relations between *A1* and *A2*, can be described as by $R(A1_{\alpha }^{-},A2_{\beta }^{-})$,$R(A1_{\alpha }^{-},A2_{\beta }^{o})$, $R(A1_{\alpha }^{o},A2_{\beta }^{-})$, and $R(A1_{\alpha }^{o},A2_{\beta }^{o})$, which can be computed by 4IM [11], 9IM [36], or other existing models.

Corresponding to conditions (ii), each topological relation between two simple crisp region $R(A1_{\alpha }^{-},A2_{\beta }^{-})$,$R(A1_{\alpha }^{-},A2_{\beta }^{o})$, $R(A1_{\alpha }^{o},A2_{\beta }^{-})$ and $R(A1_{\alpha }^{o},A2_{\beta }^{o})$ can be any one of the eight basic topological relations. Hence in theory, $R(A1_{\alpha }^{-},A2_{\beta }^{-})$,$R(A1_{\alpha }^{-},A2_{\beta }^{o})$, $R(A1_{\alpha }^{o},A2_{\beta }^{-})$, and $R(A1_{\alpha }^{o},A2_{\beta }^{o})$ can determine 8^4^ = 4096 topological relations, but only 152 basic relations for 2-dimensional simple regions in the physical world have been defined, and the relevant topological relations between *A1* and *A2* are included within the Supporting Information (S2 Table).

1. $IfR(A1_{\alpha }^{-},A2_{\beta }^{-})=disjoint\text{,}$ $R(A1_{\alpha }^{-},A2_{\beta }^{o})=R(A1_{\alpha }^{o},A2_{\beta }^{-})=R(A1_{\alpha }^{o},A2_{\beta }^{o})=disjoint\text{;}$
2. $IfR(A1_{\alpha }^{-},A2_{\beta }^{-})=meet\text{,}$ $R(A1_{\alpha }^{-},A2_{\beta }^{o})=R(A1_{\alpha }^{o},A2_{\beta }^{-})=R(A1_{\alpha }^{o},A2_{\beta }^{o})=disjoint\text{;}$
3. $IfR(A1_{\alpha }^{-},A2_{\beta }^{-})=overlap\text{,}$ $R(A1_{\alpha }^{-},A2_{\beta }^{o})=disjoint\text{,}$ $R(A1_{\alpha }^{o},A2_{\beta }^{-})=\left\{disjoint,meet,overlap,covered-by,inside\right\}\text{,}$ $R(A1_{\alpha }^{o},A2_{\beta }^{o})=disjoint\text{;}$
4. $IfR(A1_{\alpha }^{-},A2_{\beta }^{-})=overlap\text{,}$ $R(A1_{\alpha }^{-},A2_{\beta }^{o})=meet\text{,}$ $R(A1_{\alpha }^{o},A2_{\beta }^{-})=\left\{disjoint,meet,overlap,covered-by,inside\right\}\text{,}$ $\text{and }R(A1_{\alpha }^{o},A2_{\beta }^{o})=disjoint;$
5. $IfR(A1_{\alpha }^{-},A2_{\beta }^{-})=overlap$,$R(A1_{\alpha }^{-},A2_{\beta }^{o})=overlap$, $R(A1_{\alpha }^{o},A2_{\beta }^{-})=disjoint$, $R(A1_{\alpha }^{o},A2_{\beta }^{o})=disjoint$;
6. $IfR(A1_{\alpha }^{-},A2_{\beta }^{-})=overlap\text{,}$ $R(A1_{\alpha }^{-},A2_{\beta }^{o})=overlap\text{,}$ $R(A1_{\alpha }^{o},A2_{\beta }^{-})=meet\text{,}$ $R(A1_{\alpha }^{o},A2_{\beta }^{o})=disjoint\text{;}$
7. $IfR(A1_{\alpha }^{-},A2_{\beta }^{-})=overlap\text{,}$ $R(A1_{\alpha }^{-},A2_{\beta }^{o})=overlap\text{,}$ $R(A1_{\alpha }^{o},A2_{\beta }^{-})=overlap\text{,}$ $R(A1_{\alpha }^{o},A2_{\beta }^{o})=\left\{disjoint,meet,overlap\right\}\text{;}$
8. $IfR(A1_{\alpha }^{-},A2_{\beta }^{-})=overlap\text{,}$ $R(A1_{\alpha }^{-},A2_{\beta }^{o})=overlap\text{,}$ $R(A1_{\alpha }^{o},A2_{\beta }^{-})=covered-by\text{,}$ $R(A1_{\alpha }^{o},A2_{\beta }^{o})=\left\{disjoint,meet,overlap\right\}\text{;}$
9. $IfR(A1_{\alpha }^{-},A2_{\beta }^{-})=overlap\text{,}$ $R(A1_{\alpha }^{-},A2_{\beta }^{o})=overlap\text{,}$ $R(A1_{\alpha }^{o},A2_{\beta }^{-})=inside\text{,}$ $R(A1_{\alpha }^{o},A2_{\beta }^{o})=\left\{disjoint,meet,overlap,covered-by,inside\right\}\text{;}$
10. $IfR(A1_{\alpha }^{-},A2_{\beta }^{-})=overlap\text{,}$ $R(A1_{\alpha }^{-},A2_{\beta }^{o})=cover\text{,}$ $R(A1_{\alpha }^{o},A2_{\beta }^{-})=disjoint\text{,}$ $R(A1_{\alpha }^{o},A2_{\beta }^{o})=disjoint\text{;}$
11. $IfR(A1_{\alpha }^{-},A2_{\beta }^{-})=overlap\text{,}$ $R(A1_{\alpha }^{-},A2_{\beta }^{o})=cover\text{,}$ $R(A1_{\alpha }^{o},A2_{\beta }^{-})=meet\text{,}$ $R(A1_{\alpha }^{o},A2_{\beta }^{o})=disjoint\text{;}$
12. $IfR(A1_{\alpha }^{-},A2_{\beta }^{-})=overlap\text{,}$ $R(A1_{\alpha }^{-},A2_{\beta }^{o})=cover\text{,}$ $R(A1_{\alpha }^{o},A2_{\beta }^{-})=overlap\text{,}$ $R(A1_{\alpha }^{o},A2_{\beta }^{o})=\left\{disjoint,meet,overlap\right\}\text{;}$
13. $IfR(A1_{\alpha }^{-},A2_{\beta }^{-})=overlap\text{.}$ $R(A1_{\alpha }^{-},A2_{\beta }^{o})=cover\text{,}$ $R(A1_{\alpha }^{o},A2_{\beta }^{-})=covered-by\text{,}$ $R(A1_{\alpha }^{o},A2_{\beta }^{o})=\left\{disjoint,meet,overlap\right\}\text{;}$
14. $IfR(A1_{\alpha }^{-},A2_{\beta }^{-})=overlap\text{,}$ $R(A1_{\alpha }^{-},A2_{\beta }^{o})=cover\text{,}$ $R(A1_{\alpha }^{o},A2_{\beta }^{-})=inside\text{,}$ $R(A1_{\alpha }^{o},A2_{\beta }^{o})=\left\{disjoint,meet,overlap,covered-by,inside\right\}\text{;}$
15. $IfR(A1_{\alpha }^{-},A2_{\beta }^{-})=overlap\text{,}$ $R(A1_{\alpha }^{-},A2_{\beta }^{o})=contain\text{,}$ $R(A1_{\alpha }^{o},A2_{\beta }^{-})=disjoint\text{,}$ $R(A1_{\alpha }^{o},A2_{\beta }^{o})=disjoint\text{;}$
16. $IfR(A1_{\alpha }^{-},A2_{\beta }^{-})=overlap\text{,}$ $R(A1_{\alpha }^{-},A2_{\beta }^{o})=contain\text{,}$ $R(A1_{\alpha }^{o},A2_{\beta }^{-})=meet\text{,}$ $R(A1_{\alpha }^{o},A2_{\beta }^{o})=disjoint\text{;}$
17. $IfR(A1_{\alpha }^{-},A2_{\beta }^{-})=overlap\text{,}$ $R(A1_{\alpha }^{-},A2_{\beta }^{o})=contain\text{,}$ $R(A1_{\alpha }^{o},A2_{\beta }^{-})=overlap\text{,}$ $R(A1_{\alpha }^{o},A2_{\beta }^{o})=\left\{disjoint,meet,overlap,cover,contains\right\}\text{;}$
18. $IfR(A1_{\alpha }^{-},A2_{\beta }^{-})=overlap\text{,}$ $R(A1_{\alpha }^{-},A2_{\beta }^{o})=contain\text{,}$ $R(A1_{\alpha }^{o},A2_{\beta }^{-})=covered-by\text{,}$ $R(A1_{\alpha }^{o},A2_{\beta }^{o})=\left\{disjoint,meet,overlap,cover,contains\right\};$
19. $IfR(A1_{\alpha }^{-},A2_{\beta }^{-})=overlap\text{,}$ $R(A1_{\alpha }^{-},A2_{\beta }^{o})=contain\text{,}$ $R(A1_{\alpha }^{o},A2_{\beta }^{-})=inside\text{,}$ $R(A1_{\alpha }^{o},A2_{\beta }^{o})=\left\{disjoint,meet,overlap,cover,contain,covered-by,inside,equal\right\}\text{;}$
20. $IfR(A1_{\alpha }^{-},A2_{\beta }^{-})=cover\text{,}$ $R(A1_{\alpha }^{-},A2_{\beta }^{o})=contain\text{,}$ $R(A1_{\alpha }^{o},A2_{\beta }^{-})=disjoint\text{,}$ $R(A1_{\alpha }^{o},A2_{\beta }^{o})=disjoint\text{;}$
21. $IfR(A1_{\alpha }^{-},A2_{\beta }^{-})=cover\text{,}$ $R(A1_{\alpha }^{-},A2_{\beta }^{o})=contain\text{,}$ $R(A1_{\alpha }^{o},A2_{\beta }^{-})=meet\text{,}$ $R(A1_{\alpha }^{o},A2_{\beta }^{o})=disjoint\text{;}$
22. $IfR(A1_{\alpha }^{-},A2_{\beta }^{-})=cover\text{,}$ $R(A1_{\alpha }^{-},A2_{\beta }^{o})=contain\text{,}$ $R(A1_{\alpha }^{o},A2_{\beta }^{-})=overlap\text{,}$ $R(A1_{\alpha }^{o},A2_{\beta }^{o})=\left\{disjoint,meet,overlap,cover,contain\right\}\text{;}$
23. $IfR(A1_{\alpha }^{-},A2_{\beta }^{-})=cover\text{,}$ $R(A1_{\alpha }^{-},A2_{\beta }^{o})=contain\text{,}$ $R(A1_{\alpha }^{o},A2_{\beta }^{-})=covered-by\text{,}$ $R(A1_{\alpha }^{o},A2_{\beta }^{o})=\left\{disjoint,meet,overlap,cover,contain\right\}\text{;}$
24. $IfR(A1_{\alpha }^{-},A2_{\beta }^{-})=cover\text{,}$ $R(A1_{\alpha }^{-},A2_{\beta }^{o})=contain\text{,}$,$R(A1_{\alpha }^{o},A2_{\beta }^{-})=inside\text{,}$, $R(A1_{\alpha }^{o},A2_{\beta }^{o})=\left\{disjoint,meet,overlap,cover,contain,covered-by,inside,equal\right\}\text{;}$
25. $IfR(A1_{\alpha }^{-},A2_{\beta }^{-})=covered-by\text{,}$ $R(A1_{\alpha }^{-},A2_{\beta }^{o})=disjoint\text{,}$ $R(A1_{\alpha }^{o},A2_{\beta }^{-})=inside\text{,}$ $R(A1_{\alpha }^{o},A2_{\beta }^{o})=disjoint\text{;}$
26. $IfR(A1_{\alpha }^{-},A2_{\beta }^{-})=covered-by\text{,}$ $R(A1_{\alpha }^{-},A2_{\beta }^{o})=meet\text{,}$ $R(A1_{\alpha }^{o},A2_{\beta }^{-})=inside\text{,}$ $R(A1_{\alpha }^{o},A2_{\beta }^{o})=disjoint\text{;}$
27. $IfR(A1_{\alpha }^{-},A2_{\beta }^{-})=covered-by\text{,}$ $R(A1_{\alpha }^{-},A2_{\beta }^{o})=overlap\text{,}$ $R(A1_{\alpha }^{o},A2_{\beta }^{-})=inside\text{,}$ $R(A1_{\alpha }^{o},A2_{\beta }^{o})=\left\{disjoint,meet,overlap,covered-by,inside\right\}\text{;}$
28. $IfR(A1_{\alpha }^{-},A2_{\beta }^{-})=covered-by\text{,}$ $R(A1_{\alpha }^{-},A2_{\beta }^{o})=cover\text{,}$ $R(A1_{\alpha }^{o},A2_{\beta }^{-})=inside\text{,}$ $R(A1_{\alpha }^{o},A2_{\beta }^{o})=\left\{disjoint,meet,overlap,covered-by,inside\right\}\text{;}$
29. $IfR(A1_{\alpha }^{-},A2_{\beta }^{-})=covered-by\text{,}$ $R(A1_{\alpha }^{-},A2_{\beta }^{o})=contain\text{,}$ $R(A1_{\alpha }^{o},A2_{\beta }^{-})=inside\text{,}$ $R(A1_{\alpha }^{o},A2_{\beta }^{o})=\left\{disjoint,meet,overlap,cover,contain,covered-by,inside,equal\right\}\text{;}$
30. $IfR(A1_{\alpha }^{-},A2_{\beta }^{-})=contain\text{,}$ $R(A1_{\alpha }^{-},A2_{\beta }^{o})=contain\text{,}$ $R(A1_{\alpha }^{o},A2_{\beta }^{-})=disjoint\text{,}$ $R(A1_{\alpha }^{o},A2_{\beta }^{o})=disjoint\text{;}$
31. $IfR(A1_{\alpha }^{-},A2_{\beta }^{-})=contain\text{,}$ $R(A1_{\alpha }^{-},A2_{\beta }^{o})=contain\text{,}$ $R(A1_{\alpha }^{o},A2_{\beta }^{-})=meet\text{,}$ $R(A1_{\alpha }^{o},A2_{\beta }^{o})=disjoint\text{;}$
32. $IfR(A1_{\alpha }^{-},A2_{\beta }^{-})=contain\text{,}$ $R(A1_{\alpha }^{-},A2_{\beta }^{o})=contain\text{,}$ $R(A1_{\alpha }^{o},A2_{\beta }^{-})=overlap\text{,}$ $R(A1_{\alpha }^{o},A2_{\beta }^{o})=\left\{disjoint,meet,overlap,cover,contain\right\}\text{;}$
33. $IfR(A1_{\alpha }^{-},A2_{\beta }^{-})=contain\text{,}$ $R(A1_{\alpha }^{-},A2_{\beta }^{o})=contain\text{,}$ $R(A1_{\alpha }^{o},A2_{\beta }^{-})=cover\text{,}$ $R(A1_{\alpha }^{o},A2_{\beta }^{o})=contain\text{;}$
34. $IfR(A1_{\alpha }^{-},A2_{\beta }^{-})=contain\text{,}$ $R(A1_{\alpha }^{-},A2_{\beta }^{o})=contain\text{,}$ $R(A1_{\alpha }^{o},A2_{\beta }^{-})=covered-by\text{,}$ $R(A1_{\alpha }^{o},A2_{\beta }^{o})=\left\{disjoint,meet,overlap,cover,contain\right\}\text{;}$
35. $IfR(A1_{\alpha }^{-},A2_{\beta }^{-})=contain\text{,}$ $R(A1_{\alpha }^{-},A2_{\beta }^{o})=contain\text{,}$ $R(A1_{\alpha }^{o},A2_{\beta }^{-})=contain\text{,}$ $R(A1_{\alpha }^{o},A2_{\beta }^{o})=contain\text{;}$
36. $IfR(A1_{\alpha }^{-},A2_{\beta }^{-})=contain\text{,}$ $R(A1_{\alpha }^{-},A2_{\beta }^{o})=contain\text{,}$ $R(A1_{\alpha }^{o},A2_{\beta }^{-})=inside\text{,}$ $R(A1_{\alpha }^{o},A2_{\beta }^{o})=\left\{disjoint,meet,overlap,cover,contain,covered-by,inside,equal\right\}\text{;}$
37. $IfR(A1_{\alpha }^{-},A2_{\beta }^{-})=contain\text{,}$ $R(A1_{\alpha }^{-},A2_{\beta }^{o})=contain\text{,}$ $R(A1_{\alpha }^{o},A2_{\beta }^{-})=equal\text{,}$ $R(A1_{\alpha }^{o},A2_{\beta }^{o})=contain\text{;}$
38. $IfR(A1_{\alpha }^{-},A2_{\beta }^{-})=inside\text{,}$ $R(A1_{\alpha }^{-},A2_{\beta }^{o})=inside\text{,}$ $R(A1_{\alpha }^{o},A2_{\beta }^{-})=disjoint\text{,}$ $R(A1_{\alpha }^{o},A2_{\beta }^{o})=disjoint\text{;}$
39. $IfR(A1_{\alpha }^{-},A2_{\beta }^{-})=inside\text{,}$ $R(A1_{\alpha }^{-},A2_{\beta }^{o})=inside\text{,}$ $R(A1_{\alpha }^{o},A2_{\beta }^{-})=meet\text{,}$ $R(A1_{\alpha }^{o},A2_{\beta }^{o})=disjoint\text{;}$
40. $IfR(A1_{\alpha }^{-},A2_{\beta }^{-})=inside\text{,}$ $R(A1_{\alpha }^{-},A2_{\beta }^{o})=inside\text{,}$ $R(A1_{\alpha }^{o},A2_{\beta }^{-})=overlap\text{,}$ $R(A1_{\alpha }^{o},A2_{\beta }^{o})=\left\{disjoint,meet,overlap,covered-by,inside\right\}\text{;}$
41. $IfR(A1_{\alpha }^{-},A2_{\beta }^{-})=inside\text{,}$ $R(A1_{\alpha }^{-},A2_{\beta }^{o})=inside\text{,}$ $R(A1_{\alpha }^{o},A2_{\beta }^{-})=cover\text{,}$ $R(A1_{\alpha }^{o},A2_{\beta }^{o})=\left\{disjoint,meet,overlap,covered-by,inside\right\}\text{;}$
42. $IfR(A1_{\alpha }^{-},A2_{\beta }^{-})=inside\text{,}$ $R(A1_{\alpha }^{-},A2_{\beta }^{o})=inside\text{,}$ $R(A1_{\alpha }^{o},A2_{\beta }^{-})=contain\text{,}$ $R(A1_{\alpha }^{o},A2_{\beta }^{o})=\left\{disjoint,meet,overlap,cover,contain,covered-by,inside,equal\right\}\text{;}$
43. $IfR(A1_{\alpha }^{-},A2_{\beta }^{-})=inside\text{,}$ $R(A1_{\alpha }^{-},A2_{\beta }^{o})=inside\text{,}$ $R(A1_{\alpha }^{o},A2_{\beta }^{-})=covered-by\text{,}$ $R(A1_{\alpha }^{o},A2_{\beta }^{o})=inside\text{;}$
44. $IfR(A1_{\alpha }^{-},A2_{\beta }^{-})=inside\text{,}$ $R(A1_{\alpha }^{-},A2_{\beta }^{o})=inside\text{,}$ $R(A1_{\alpha }^{o},A2_{\beta }^{-})=inside\text{,}$ $R(A1_{\alpha }^{o},A2_{\beta }^{o})=inside\text{;}$
45. $IfR(A1_{\alpha }^{-},A2_{\beta }^{-})=inside\text{,}$ $R(A1_{\alpha }^{-},A2_{\beta }^{o})=inside\text{,}$ $R(A1_{\alpha }^{o},A2_{\beta }^{-})=equal\text{,}$ $R(A1_{\alpha }^{o},A2_{\beta }^{o})=inside\text{;}$
46. $IfR(A1_{\alpha }^{-},A2_{\beta }^{-})=equal\text{,}$ $R(A1_{\alpha }^{-},A2_{\beta }^{o})=R(A1_{\alpha }^{o},A2_{\beta }^{-})=contain\text{,}$ $R(A1_{\alpha }^{o},A2_{\beta }^{o})=\left\{disjoint,meet,overlap,cover,contain,covered-by,inside,equal\right\}\text{.}$

## Comparison with Existing Models

In dealing with fuzzy spatial objects, Cohn and Gotts [15] proposed the ‘egg-yolk’ model with two concentric sub-regions, indicating the degree of ‘membership’ in a vague/fuzzy region. In this model, the ‘yolk’ represents the precise part and ‘egg’ represents the vague/fuzzy part of a region. The ‘egg-yolk’ model is an extension of RCC theory [17] into the vague/fuzzy region, a total of forty-six relations can be identified [15]. Based on the 9-intersection model (9IM, Eq. (1)), Clemmentini and Di Felice defined a region with a broad boundary, by using two simple regions [18, 20], this broad boundary is denoted by Δ*A*. Based on empty and non-empty invariance, Clementini and Di Felice’s Algebraic Model as equation (2):

$$R_{9}\left(A,B\right)=\left[\begin{array}{l} \begin{array}{cc} \begin{array}{cc} A^{{}^{\circ}}\cap B^{{}^{\circ}} & A^{{}^{\circ}}\cap \Delta B \end{array} & A^{{}^{\circ}}\cap B^{-} \end{array}\\ \begin{array}{ccc} \begin{array}{l} \Delta A\cap B^{{}^{\circ}}\\ A^{-}\cap B^{{}^{\circ}} \end{array} & \begin{array}{l} \Delta A\cap \Delta B\\ A^{-}\cap \Delta B \end{array} & \begin{array}{l} \Delta A\cap B^{-}\\ A^{-}\cap B^{-} \end{array} \end{array} \end{array}\right]$$ (2)

Equation (2) gave a total of forty-four relations between two spatial regions with a broad boundary. The extended 9-intersection model (Eq. (2)) substantially agrees with the ‘egg-yolk’ model, though the former removes two relations considered as invalid in the geographical environment.

**Fig 5:**
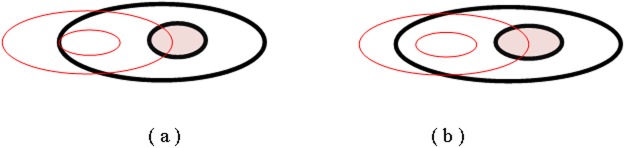
Two different topological relations between *A1* and *A2*.

For example, in the table 2 (18) and (21), as shown in Fig. 5(a, b), the equation (2) yielded the same matrix, $R_{9}(A,B)=\left[\begin{array}{l} \begin{array}{ccc} 0 & 1 & 1 \end{array}\\ \begin{array}{ccc} 1 & 1 & 1 \end{array}\\ \begin{array}{ccc} 0 & 1 & 1 \end{array} \end{array}\right]$. That is to say, the topological relations are same, but they are obviously different from each other as shown in Fig. 5 (a, b). And then, we'll discuss the topological relations (as shown in Fig. 5) of using the new combinational reasoning method in this paper.

Fuzzy topology is dependent on the value of *α*or*β* used in leveling cuts. Different values of *α*and*β* generate different fuzzy topologies and may have different topological structures. Therefore, we can generate the suitable fuzzy topology by adjusting the value of*α*or*β*, and the generated fuzzy topology can thus match the cases of the application concerned. Moreover, different values of *α*and*β*can provide a multi-directional spatial analysis in GIS [29]. Details about calculating the value of *α*or*β* in fuzzy topology and fuzzy sets, we can see the relevant literatures [27, 29]. In this paper, based on the research of Liu and Shi, we propose a new definition for simple fuzzy line segments and simple fuzzy regions, and also propose a new combinational reasoning method to compute the fuzzy topological relations between two simple spatial regions. Therefore, it’s important to note that the values of*α*and*β*in the Fig. 5 only used to demonstrate the validity of the new method, in the actual application and analysis, an optimal value of*α*or*β*can be obtained by investigating these fuzzy topologies, and generated a suitable fuzzy topological relations [29]. We can generate a suitable fuzzy topology by adjusting the value of *α*or*β*, and then more information can be generated for spatial queries by applying fuzzy topology.

In Fig. 5 (a) and (b), the simple fuzzy region *A1* for given*α*, *A2* for given*β*, the topological relation between *A1* and *A2* can be obtained by calculating $R(A1_{\alpha }^{-},A2_{\beta }^{-})$, $R(A1_{\alpha }^{-},A2_{\beta }^{o})$, $R(A1_{\alpha }^{o},A2_{\beta }^{-})$, and $R(A1_{\alpha }^{o},A2_{\beta }^{o})$.

For Fig. 5 (a), we used 9IM (Eq. (1)) to compute $R(A1_{\alpha }^{-},A2_{\beta }^{-})$, $R(A1_{\alpha }^{-},A2_{\beta }^{o})$, $R(A1_{\alpha }^{o},A2_{\beta }^{-})$, and $R(A1_{\alpha }^{o},A2_{\beta }^{o})$, so the topological relation can be described as follows:

a): $R(A1_{\alpha }^{-},A2_{\beta }^{-})=overlap\text{,}$ $R(A1_{\alpha }^{-},A2_{\beta }^{o})=overlap\text{,}$ $R(A1_{\alpha }^{o},A2_{\beta }^{-})=covered-by\text{,}$ $\text{and }R(A1_{\alpha }^{o},A2_{\beta }^{o})=disjoint\text{;}$

$$R(A1_{\alpha }^{-},A2_{\beta }^{-})=\left[\begin{array}{l} \begin{array}{ccc} 1 & 1 & 1 \end{array}\\ \begin{array}{ccc} 1 & 1 & 1 \end{array}\\ \begin{array}{ccc} 1 & 1 & 1 \end{array} \end{array}\right]\text{,}$$

$$R(A1_{\alpha }^{-},A2_{\beta }^{o})=\left[\begin{array}{l} \begin{array}{ccc} 1 & 1 & 1 \end{array}\\ \begin{array}{ccc} 1 & 1 & 1 \end{array}\\ \begin{array}{ccc} 1 & 1 & 1 \end{array} \end{array}\right]\text{,}$$

$$R(A1_{\alpha }^{o},A2_{\beta }^{-})=\left[\begin{array}{l} \begin{array}{ccc} 1 & 0 & 0 \end{array}\\ \begin{array}{ccc} 1 & 1 & 0 \end{array}\\ \begin{array}{ccc} 1 & 1 & 1 \end{array} \end{array}\right]\text{,}$$

$$R(A1_{\alpha }^{o},A2_{\beta }^{o})=\left[\begin{array}{l} \begin{array}{ccc} 0 & 0 & 1 \end{array}\\ \begin{array}{ccc} 0 & 0 & 1 \end{array}\\ \begin{array}{ccc} 1 & 1 & 1 \end{array} \end{array}\right]\text{;}$$

For Fig. 5 (b), we used 9IM (Eq. (1)) to compute $R(A1_{\alpha }^{-},A2_{\beta }^{-})$, $R(A1_{\alpha }^{-},A2_{\beta }^{o})$, $R(A1_{\alpha }^{o},A2_{\beta }^{-})$, and $R(A1_{\alpha }^{o},A2_{\beta }^{o})$, so the topological relation can be described as follows:

b): $R(A1_{\alpha }^{-},A2_{\beta }^{-})=overlap\text{,}$ $R(A1_{\alpha }^{-},A2_{\beta }^{o})=overlap\text{,}$ $R(A1_{\alpha }^{o},A2_{\beta }^{-})=inside\text{,}$ $\text{and }R(A1_{\alpha }^{o},A2_{\beta }^{o})=disjoint\text{;}$

$$R(A1_{\alpha }^{-},A2_{\beta }^{-})=\left[\begin{array}{l} \begin{array}{ccc} 1 & 1 & 1 \end{array}\\ \begin{array}{ccc} 1 & 1 & 1 \end{array}\\ \begin{array}{ccc} 1 & 1 & 1 \end{array} \end{array}\right]\text{,}$$

$$R(A1_{\alpha }^{-},A2_{\beta }^{o})=\left[\begin{array}{l} \begin{array}{ccc} 1 & 1 & 1 \end{array}\\ \begin{array}{ccc} 1 & 1 & 1 \end{array}\\ \begin{array}{ccc} 1 & 1 & 1 \end{array} \end{array}\right]\text{,}$$

$$R(A1_{\alpha }^{o},A2_{\beta }^{-})=\left[\begin{array}{l} \begin{array}{ccc} 1 & 0 & 0 \end{array}\\ \begin{array}{ccc} 1 & 0 & 0 \end{array}\\ \begin{array}{ccc} 1 & 1 & 1 \end{array} \end{array}\right]\text{,}$$

$$R(A1_{\alpha }^{o},A2_{\beta }^{o})=\left[\begin{array}{l} \begin{array}{ccc} 0 & 0 & 1 \end{array}\\ \begin{array}{ccc} 0 & 0 & 1 \end{array}\\ \begin{array}{ccc} 1 & 1 & 1 \end{array} \end{array}\right]\text{.}$$

Through the above comparison analysis, the new proposed combinational reasoning method (when taking different values of*α*and*β*) not only can obtain the topological relations of simple fuzzy regions as listed in existing studies [15, 16, 18, 20], but also the topological relations not currently listed.

## Conclusion

Fuzzy topological relations between simple spatial objects can be used for fuzzy spatial queries, fuzzy spatial analyses, and other questions. This paper presented a combinational reasoning method of quantitative fuzzy topological relations for simple spatial regions in GIS. Based on the research of Liu and Shi, we proposed a new definition for simple fuzzy line segments and simple fuzzy regions based on the computational fuzzy topology, we also proposed a new combinational reasoning method to compute the fuzzy topological relations between simple spatial regions, an analysis of the new method exposes: (1) 23 different topological relations between a simple crisp region and a simple fuzzy region; (2) 152 different topological relations between two simple fuzzy regions. Based on the new definitions, the proposed method in this study not only computes the topological relations between a simple crisp region and a simple fuzzy region, but also computes the topological relations between two simple fuzzy regions. In the end, we have discussed some examples to demonstrate the validity of the new method. Through comparisons of results, we showed that the proposed method can make finer distinctions, as it is more expressive than the existing fuzzy models.

## Supporting Information

S1 Table The 23 relations between a simple crisp region and a simple fuzzy region in *R* ^*2*^. (PDF)

S2 Table The 152 relations between two simple fuzzy regions in *R* ^*2*^. (PDF)
