# Supplementary material for: Combinational Reasoning of Quantitative Fuzzy Topological Relations for Simple Fuzzy Regions
Source: PLoS One. 2015 Mar 16;10(3):e0117379. doi: 10.1371/journal.pone.0117379 (PMC4361350; doi:10.1371/journal.pone.0117379)

Table 1. The 23 relations between a simple crisp region and a simple fuzzy region in $R^2$

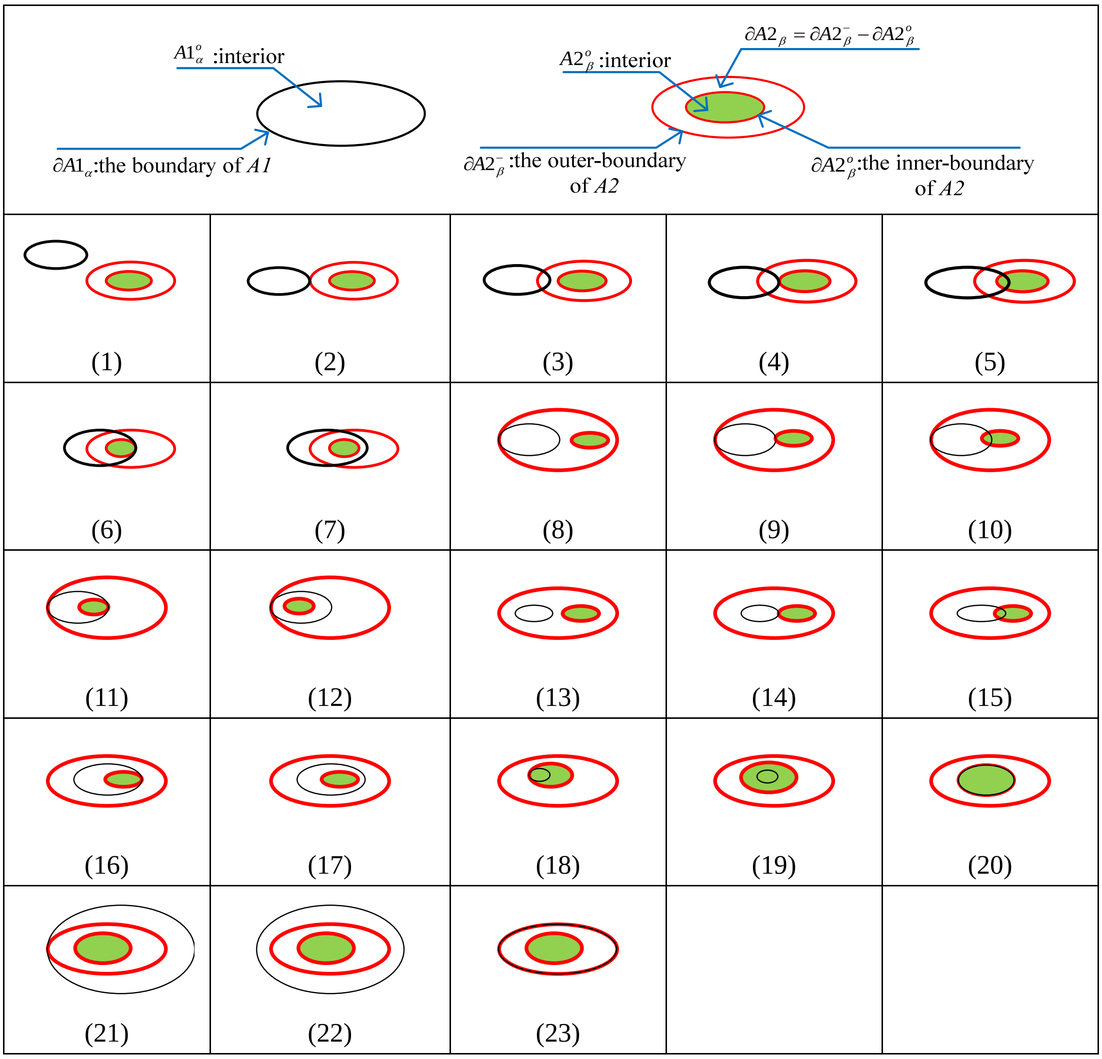

Supplement: S1 Table — (PDF) [file pone.0117379.s001.pdf]
