# Supplementary material for: Combinational Reasoning of Quantitative Fuzzy Topological Relations for Simple Fuzzy Regions
Source: PLoS One. 2015 Mar 16;10(3):e0117379. doi: 10.1371/journal.pone.0117379 (PMC4361350; doi:10.1371/journal.pone.0117379)
Supplement: S2 Table — (PDF) [file pone.0117379.s002.pdf]

Table 2. The 152 relations between two simple fuzzy regions in R^2

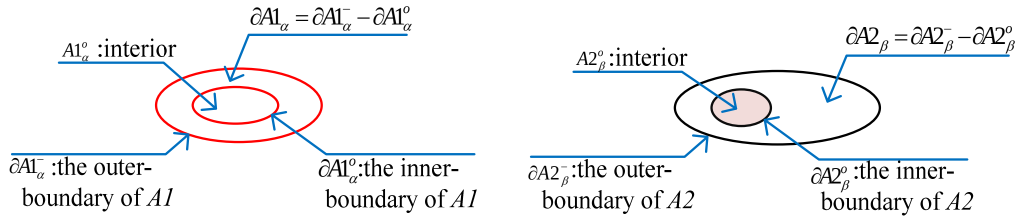

(1)	(2)	(3)	(4)	(5)
(6)	(7)	(8)	(9)	(10)
(11)	(12)	(13)	(14)	(15)
(16)	(17)	(18)	(19)	(20)
(21)	(22)	(23)	(24)	(25)
(26)	(27)	(28)	(29)	(30)
(31)	(32)	(33)	(34)	(35)
(36)	(37)	(38)	(39)	(40)
(41)	(42)	(43)	(44)	(45)
(46)	(47)	(48)	(49)	(50)
(51)	(52)	(53)	(54)	(55)

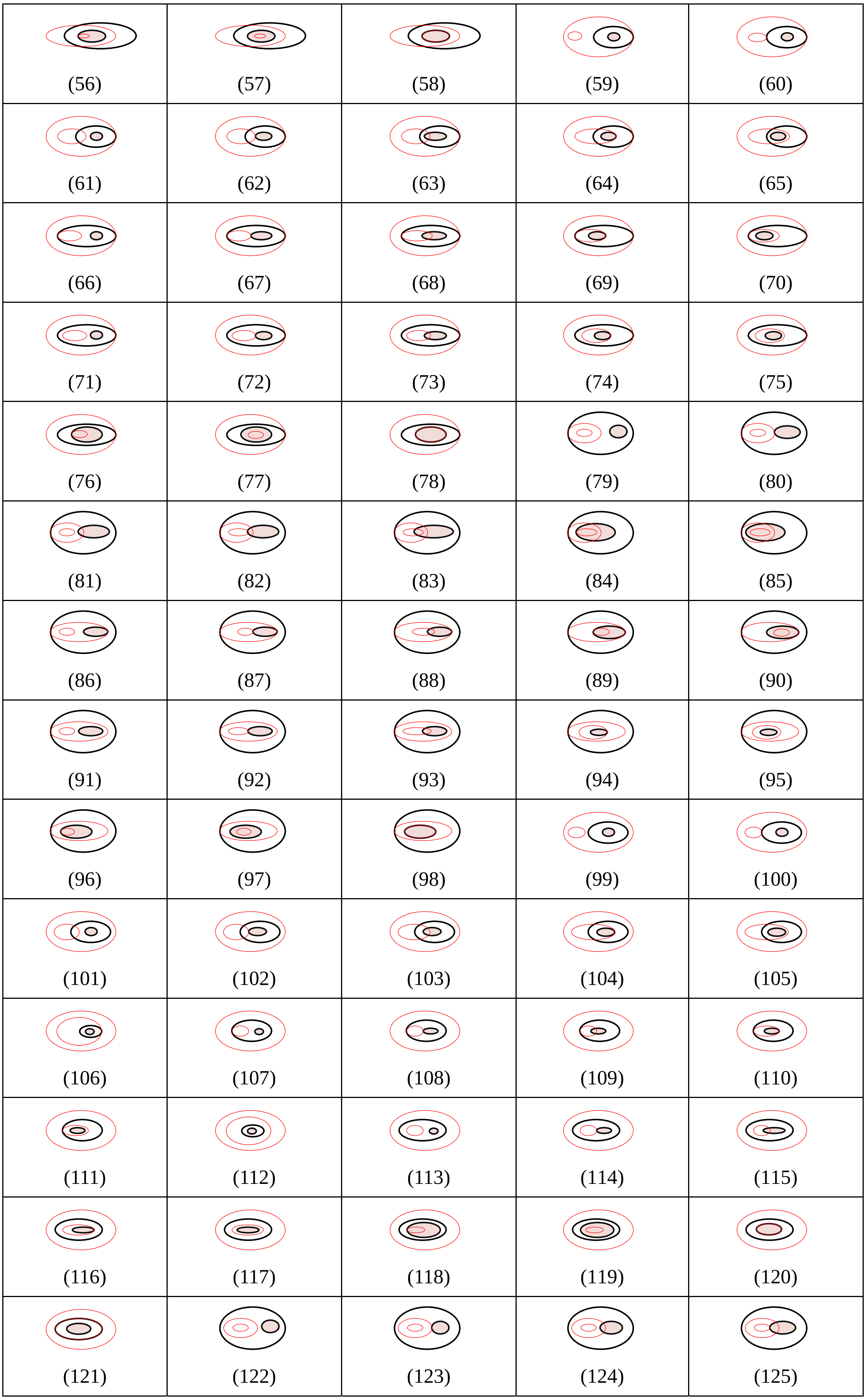
(56) (57) (58) (59) (60)
(61) (62) (63) (64) (65)
(66) (67) (68) (69) (70)
(71) (72) (73) (74) (75)
(76) (77) (78) (79) (80)
(81) (82) (83) (84) (85)
(86) (87) (88) (89) (90)
(91) (92) (93) (94) (95)
(96) (97) (98) (99) (100)
(101) (102) (103) (104) (105)
(106) (107) (108) (109) (110)
(111) (112) (113) (114) (115)
(116) (117) (118) (119) (120)
(121) (122) (123) (124) (125)

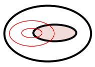 (126)	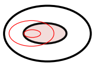 (127)	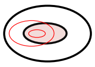 (128)	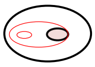 (129)	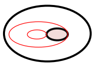 (130)
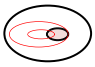 (131)	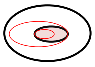 (132)	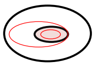 (133)	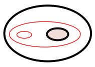 (134)	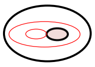 (135)
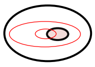 (136)	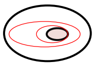 (137)	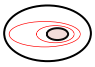 (138)	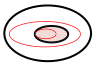 (139)	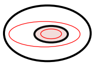 (140)
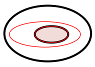 (141)	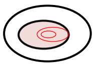 (142)	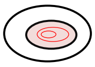 (143)	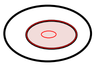 (144)	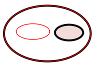 (145)
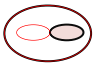 (146)	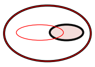 (147)	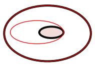 (148)	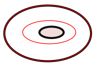 (149)	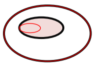 (150)
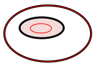 (151)	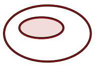 (152)			
